# Identification of Five Glycolysis-Related Gene Signature and Risk Score Model for Colorectal Cancer

**DOI:** 10.3389/fonc.2021.588811

**Published:** 2021-03-04

**Authors:** Jun Zhu, Shuai Wang, Han Bai, Ke Wang, Jun Hao, Jian Zhang, Jipeng Li

**Affiliations:** ^1^State Key Laboratory of Cancer Biology, Institute of Digestive Diseases, Xijing Hospital, The Fourth Military Medical University, Xi’an, China; ^2^Department of Radiation Oncology, Xijing Hospital, The Fourth Military Medical University, Xi’an, China; ^3^Department of Experiment Surgery, Xijing Hospital, Fourth Military Medical University, Xi’an, China; ^4^State Key Laboratory of Cancer Biology, Department of Biochemistry and Molecular Biology, The Fourth Military Medical University, Xi’an, China

**Keywords:** glycolytic gene, prognosis analysis, colorectal cancer, GPC1, ENO3, P4HA1, SPAG4, STC2

## Abstract

Metabolic changes, especially in glucose metabolism, are widely established during the occurrence and development of tumors and regarded as biological markers of pan-cancer. The well-known ‘Warburg effect’ demonstrates that cancer cells prefer aerobic glycolysis even if there is sufficient ambient oxygen. Accumulating evidence suggests that aerobic glycolysis plays a pivotal role in colorectal cancer (CRC) development. However, few studies have examined the relationship of glycolytic gene clusters with prognosis of CRC patients. Here, our aim is to build a glycolysis-associated gene signature as a biomarker for colorectal cancer. The mRNA sequencing and corresponding clinical data were downloaded from TCGA and GEO databases. Gene set enrichment analysis (GSEA) was performed, indicating that four gene clusters were significantly enriched, which revealed the inextricable relationship of CRC with glycolysis. By comparing gene expression of cancer and adjacent samples, 236 genes were identified. Univariate, multivariate, and LASSO Cox regression analyses screened out five prognostic-related genes (ENO3, GPC1, P4HA1, SPAG4, and STC2). Kaplan–Meier curves and receiver operating characteristic curves (ROC, AUC = 0.766) showed that the risk model could become an effective prognostic indicator (*P* < 0.001). Multivariate Cox analysis also revealed that this risk model is independent of age and TNM stages. We further validated this risk model in external cohorts (GES38832 and GSE39582), showing these five glycolytic genes could emerge as reliable predictors for CRC patients’ outcomes. Lastly, based on five genes and risk score, we construct a nomogram model assessed by C-index (0.7905) and calibration plot. In conclusion, we highlighted the clinical significance of glycolysis in CRC and constructed a glycolysis-related prognostic model, providing a promising target for glycolysis regulation in CRC.

## Introduction

Colorectal cancer (CRC) is the third most common malignant tumor and the second common cause of cancer-related death worldwide (1). Due to the lack of early clinical symptoms and predictive markers, CRC patients often are diagnosed at advanced stages and forfeit the occasion of surgical treatment. Patients in advanced stages especially with metastatic lesions could have a poor prognosis (2), and the 5-year survival rate of CRC patients is only 13% (3, 4). Therefore, it is crucial to discover and identify novel diagnostic indicator for the early detection and treatment of CRC.

Altered energy metabolism, which fuels tumor cell growth and proliferation, has been heralded as an emerging cancer hallmark (5, 6). In 1924, Warburg found that cancer cells behave bizarrely and cancer cells could maintain a high glycolysis rate compared with adjacent normal tissue even in the presence of oxygen; that is the well-known ‘Warburg effect’ (7, 8). Based on this discovery, (18F)-fluorodeoxyglucose-positron emission tomography (FDG-PET) is applied to scan out a tumor tissue and common metastasis sites because of its generally high uptake of the glucose analog FDG. Many glycolysis-related genes identified upregulated in CRC contains hypoxiainducible factor-1 *α* (HIF-1 *α*) (9–12), glucose transporter family (GLUT) (13–15), hexokinase (HK) (16), pyruvate kinase (PKM) (17, 18), pyruvate dehydrogenase kinases (PDK) (11) and lactate dehydrogenase A(LDHA) (12, 15). LDHA converted to lactic acid metabolic enzymes is identified as the oncogene MYC’s first metabolic target (19). Apart from glycolysis-related genes, some long non-coding RNAs (LncRNAs) were regarded as a significant role in the glycolysis–pathway. LncRNA GLCC1 stabilizes c-Myc transcriptional factor and further facilitates the expression of its target genes (such as LDHA), consequently reprogramming glycolytic metabolism for CRC proliferation (20). Another reporter found that LncRNA FEZF1-AS1 could bind and increase the stability of PKM2 protein, resulting in increased cytoplasmic and nuclear PKM2 level, which further activate STAT3 signaling (21). Therefore, glycolysis-related genes have been a potential target for cancer therapy, and many associated molecules participate in the regulation of glucose metabolism in CRC cells.

CRC is widespread and continuing progress in developing and developed countries. Another worrying phenomenon is the rise in patients diagnosed with CRC younger than 50 years old (22). Thus, there is an urgent need for improved CRC screening and therapeutics. Clinically, superior or comprehensive models to predict the overall survival (OS) of CRC patients are also needed (23). The rapid development of genomic profiling and analytical ability of big data provide great convenience for constructing risk models for cancer patients and prognostic evaluation. In this study, we built a five-gene signature based on glycolysis from The Cancer Genome Atlas (TCGA) and validated it in Gene Expression Omnibus (GEO) database. By multivariate Cox regression and survival analysis, we demonstrated that the risk model could be considered an independent CRC indicator. Collectively, our results suggested the glycolysis-related risk model may serve as a clinical outcome indicator for CRC, and we provide potential therapeutic targets for CRC patients.

## Methods

### Data Collection

The mRNA expression data and corresponding clinical features of CRC patients were downloaded from TCGA (https://portal.gdc.cancer.gov/) and Gene Expression Omnibus (GEO) (https://www.ncbi.nlm.nih.gov/geo/). There are 39 paracancerous tissues and 398 cases of cancerous tissues in the TCGA dataset, among which 379 patients had complete follow-up data. As well, there are 122 and 579 CRC patients in the GSE38832 and GSE39582, respectively (**Table S1**). Ten paired CRC samples (cancerous and adjacent tissue) were collected from the Xijing digestive hospital.

### Glycolytic Process-Related Gene Sets Enriched by GSEA

Gene set enrichment analysis (GSEA) (24) was performed with GSEA version 4.0.1 software and used to complete glycolysis-related enrichment analysis with 1,000 permutation number. Six gene sets (Kegg glycolysis pathway, Kegg glycolysis gluconeogenesis, Go glycolytic process, Hallmark glycolysis, Reactome regulation of glycolysis, Reactome glycolysis) were downloaded from the MSigDB of GSEA website (www.broadinstitute.org/gsea/) and chosen as reference gene sets. We set the cut-off criteria as gene size >=15, |normalized enrichment score (NES)| >1.5 and nominal P-value (NOM P-value) <0.05.

### Identification of Differentially Expressed Genes and Prognostic Genes

298 genes were extracted from the four gene sets enriched by GESA, and the linear models for microarray data (LIMMA) R package was performed for DEGs identified between paracancerous and cancer samples. Cut-off values were set at P value <0.05 and |log2 fold change| >zero. DEGs were associated with the OS of patients by univariable Cox analysis. P <0.05 was considered as a statistically significant difference.

### Construction of Risk Score Model by Multivariate Cox and LASSO Regression

Univariate and multivariate Cox regression analyses were used by ‘survival’ R package, and LASSO was analyzed with ‘glmnet’ package to obtain the most useful predictive genes. The risk score model was constructed based on the corresponding coefficients, and risk score of each patient was generated by multiplying the expression and coefficients. Through the median risk scores, two groups were divided (high- and low-risk subgroups). Receiver operating characteristic (ROC) curves and Kaplan–Meier curves (K–M curves) were applied to evaluate the predictive capability of the risk model. In addition, univariate and multivariate Cox regression analyses were used to explore the prognostic efficiency of the risk score models and other clinicopathological features.

### Construction and Validation of a Predictive Nomogram

A nomogram can be performed to predict the prognosis of various cancer (25). In the TCGA datasets, five glycolysis-related genes identified by multivariate Cox regression were included to build a nomogram using the ‘rms’ package in R to investigate the 3- and 5-year survival rate of CRC patients. To assess the discrimination and accuracy of the nomogram, we calculated the concordance index (C-index) and plotted a calibration curve.

### Alternation of Five Genes in the Model

The cBioPortal dataset (https://www.cbioportal.org/) possesses multi-dimensional cancer genomics data. We used it to explore the genetic alterations of five genes (TCGA-Pancancer Atlas) and their relationship with other related genes. The network of glycolytic genes and highly related genes was visualized in Cytoscape software (version 3.61).

### Immunohistochemistry and RT-PCR

Immunohistochemistry images of the five model genes were downloaded from the Human Protein Atlas (https://www.proteinatlas.org/). Messenger RNA (mRNA) of 10 paired tissues and six kinds of cell lines was extracted using TRIzol’s method (Invitrogen, Carlsbad, CA, USA), and quantitative real-time PCR was conducted with SYBR-green PCR MasterMix (TaKaRa). The study was approved by the Xijing Hospital Ethics Committee (No. KY20203269-1), and informed consent was taken from all the patients.

### Statistical Analysis

R (version 3.63, http://www.r-project.org/) was applied to perform statistical analysis. All statistical tests were bilateral, and *P <*0.05 was considered as statistically significant.

## Results

### Overall Design of the Study

**Figure 1:**
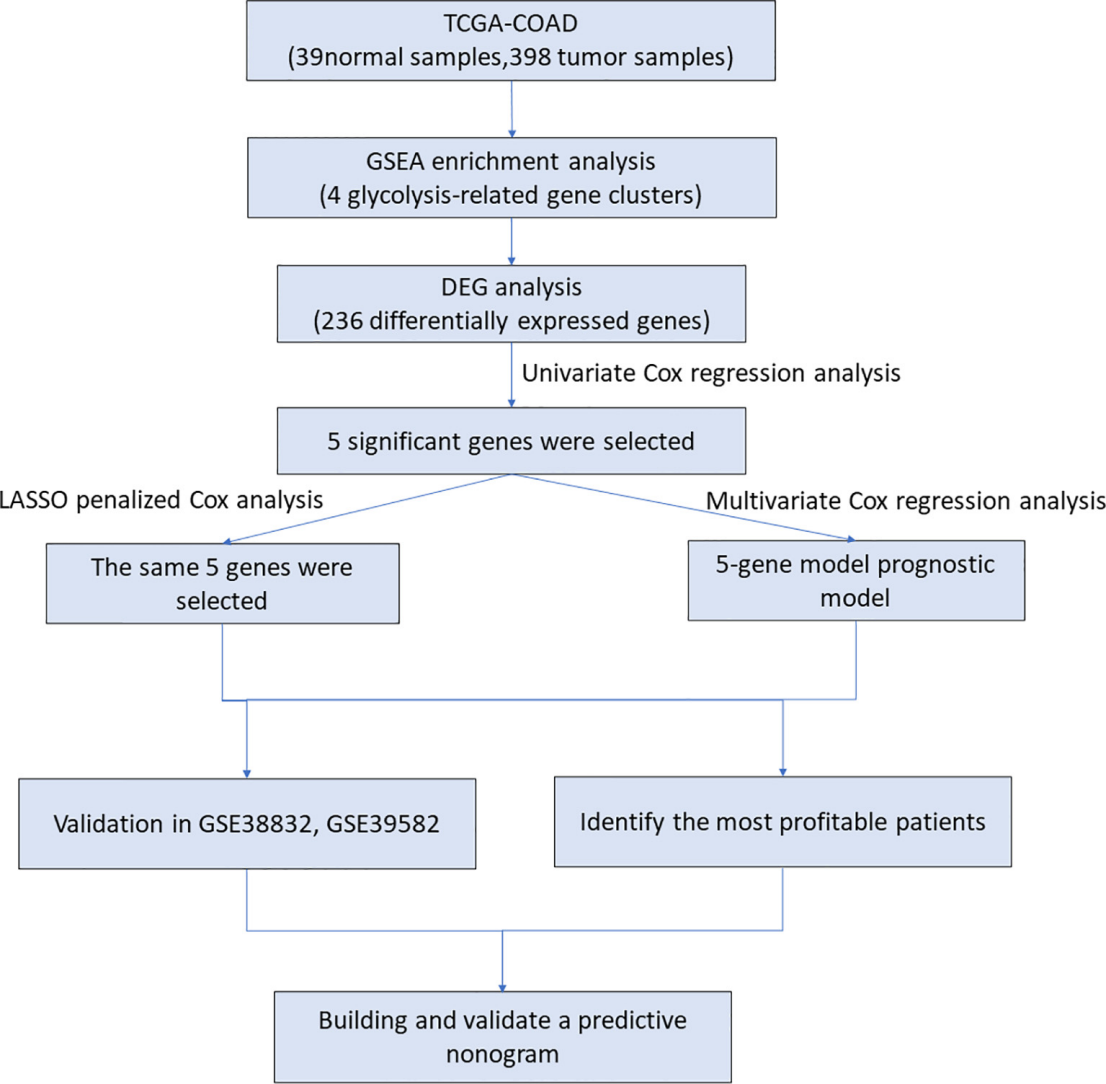
The workflow identified the glycolysis-related prognostic risk model in CRC patients. TCGA, The Cancer Genome Atlas; COAD, Colon adenocarcinoma; DEG, differentially expressed gene; GSEA, gene set enrichment analysis.

The flow chart of our research is shown in **Figure 1**. The clinical data of CRC patients and corresponding gene expression profiles were downloaded from the TCGA database. The glycolysis-related gene sets were enriched by GSEA. The risk model of glycolysis related genes was established by multivariate Cox analysis and LASSO algorithm. The five-gene risk model was validated by survival analysis and ROC curves. Patients were divided into two parts based on their clinical characteristics, and survival analysis was performed to determine which group would be predicted accurately. In addition, two external GEO data sets were downloaded to verify this model, and a nomogram based on TCGA data was built. Calibration and C-index were used to assess the nomogram.

### Four Glycolysis-Related Gene Clusters Were Significantly Enriched in CRC Patients

**Figure 2:**
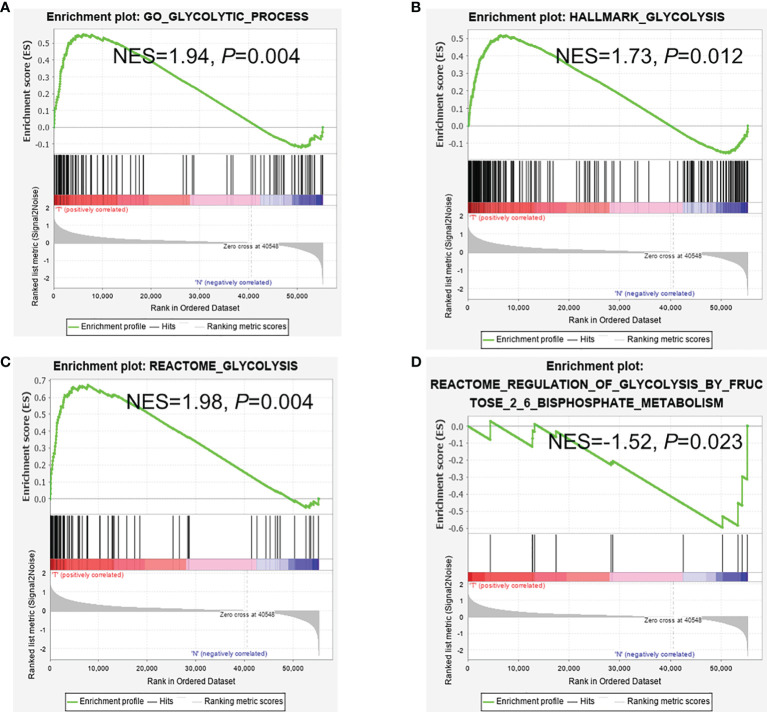
GSEA analysis between cancer and normal samples. **(A–C)** GSEA reveals that glycolysis pathways were enriched in CRC tissues and **(D)** in adjacent normal tissues. **(A)** Go glycolytic process; **(B)** Hallmark glycolysis; **(C)** Reactome glycolysis; **(D)** Reactome regulation of glycolysis.

In the TCGA-COAD dataset, 39 cases were normal samples, and 398 cases were tumor samples. Four of the six gene sets were significantly enriched (**Figure 2**). The GSEA results documented that glycolysis pathways play a pivotal role in CRC patients’ occurrence and progression. 298 genes associated with glycolysis pathway were extracted in transcriptome expression of TCGA colorectal patients for further analysis.

### Identification of DEGs in Glycolysis-Associated Clusters

These 298 glycolytic genes were analyzed for differential expression between tumor samples and non-tumor tissues. To construct a better risk model, logFC (log2 Fold change) was not strictly restricted, and we defined that gene could be retained only if |logFC| >0 and *P* <.05. The results showed that most (236) glycolytic genes were selected, and GSEA analysis was undoubtedly effective and reliable.

### Five Glycolysis-Related Genes Were Selected by Univariate Cox Analysis

**Figure 3:**
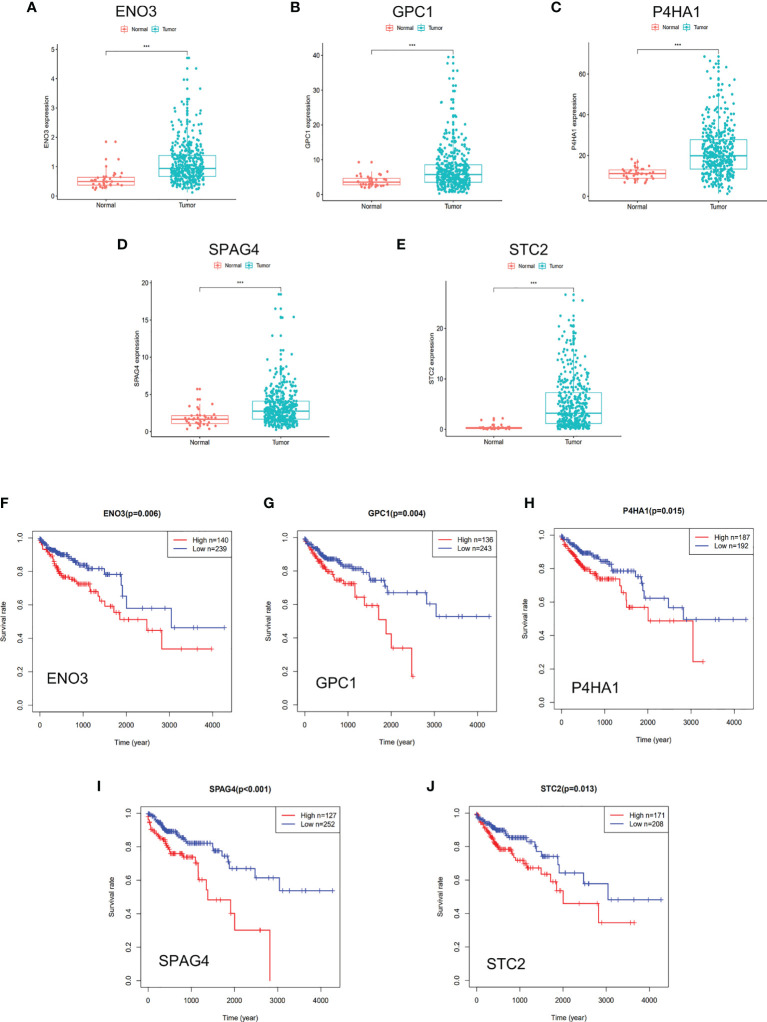
DGE and survival analysis of five glycolysis-related genes. **(A–E)** Five glycolysis-related genes were upregulated in tumor tissue than adjacent in normal tissue. **(F–J)** Kaplan–Meier curves revealed that patients with low gene expression had better outcomes than those with high expression. **(A, F)** ENO3, **(B, G)** GPC1, **(C, H)** P4HA1, **(D, I)** SPAG4, **(E, J)** STC2. *** represents P < 0.001.

Univariate Cox regression analysis was performed for preliminary selection of prognostic genes, and five genes were established when the cut-off value was P <0.05 (**Table 1**). The five genes are Enolase 3 (ENO3), Glypican-1 (GPC1), Prolyl 4-hydroxylase subunit alpha 1 (P4HA1), Sperm associated antigen 4 (SPAG4), and Stanniocalcin 2 (STC2). As is expected, these five genes are highly expressed in tumor tissues than in normal samples (**Figures 3A–E**), and patients with low gene expression had a better prognosis than those with high expression (**Figures 3F–J**). The Survminer R package was conducted to find the optimal cut-off value for distinguishing between high and low expression of these genes. These results showed these five glycolytic genes could emerge as reliable predictors for CRC patients’ outcomes and were chosen for further building risk score model.

**Table 1:** The results of univariate Cox analysis in CRC.

Gene	*β*(Cox)	HR	HR.95L	HR.95H	P value
**ENO3**	0.3989	1.490143	1.141657	1.945002	0.003339
**P4HA1**	0.0239	1.024144	1.005205	1.043441	0.012244
**GPC1**	0.02693	1.037619	1.004159	1.072195	0.027237
**SPAG4**	0.1174	1.12462	1.047549	1.20736	0.001185
**STC2**	0.0442	1.045169	1.002151	1.090033	0.039384

HR, hazard ratio; β is calculated by univariate Cox analysis.

### Construction of Risk Score Model by Multivariate and LASSO Regression Analyses

**Figure 4:**
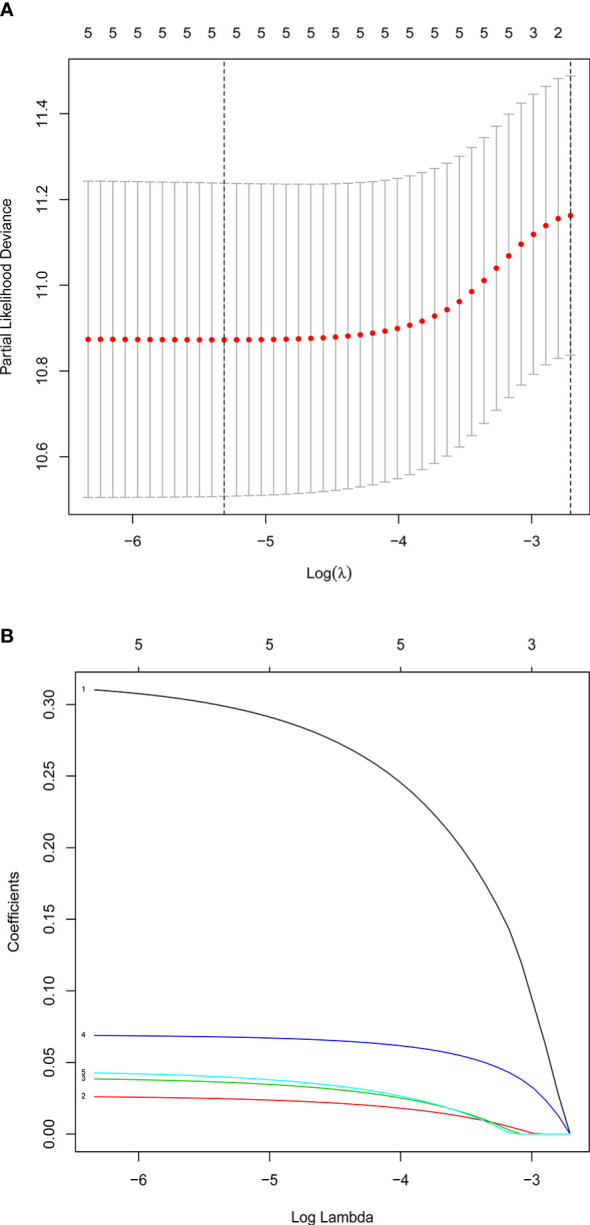
Confirmation of prognostic genes by LASSO analysis. **(A)** Distribution of LASSO coefficients for five genes. **(B)** Partial likelihood deviation of the LASSO coefficient distribution. Two vertical lines are lambda.min and lambda.lse.

**Figure 5:**
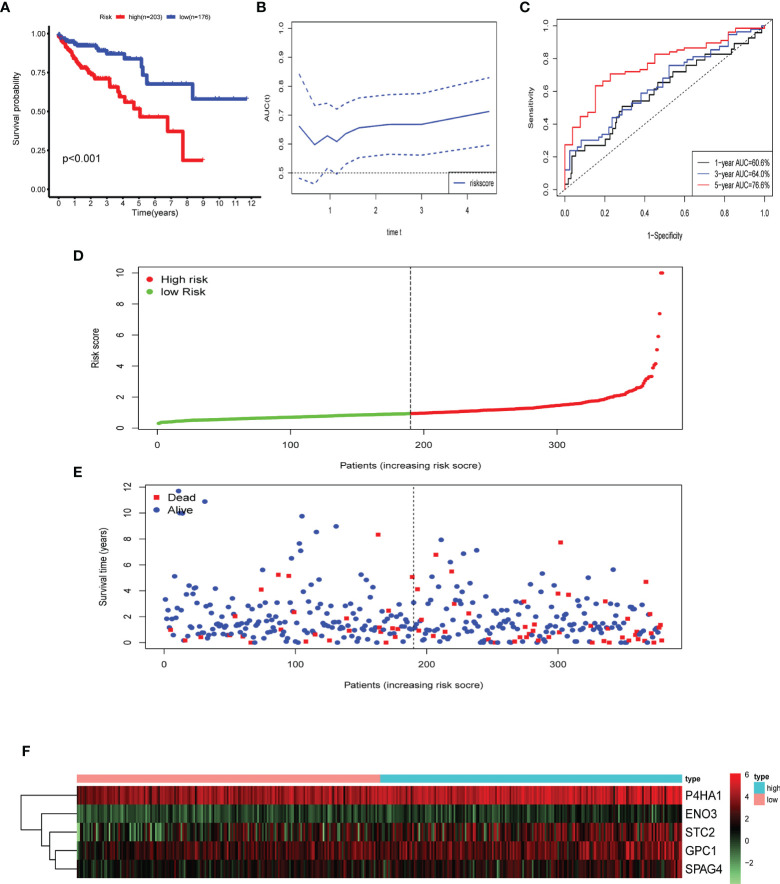
Identification of prognostic risk gene signature associated with glycolysis. **(A)** Survival analysis to verify the difference between the high- and low-risk groups. **(B, C)** Time-dependent ROC to evaluate the predictive efficacy of the risk model. **(D)** Distribution of risk scores of each CRC patient. **(E)** Correlation between survival time and survival status of each patient. **(F)** The expression pattern of five glycolysis-related genes.

Multivariate Cox and LASSO regression analyses were designed to filter the hub genes in the risk model to predict CRC patients’ outcomes. Five genes were all accepted in the risk model, and coefficients were validated by multivariate Cox analysis (strengthened by forward and backward method) (**Table 2**). The minimum Akaike information criterion (AIC) of the risk model is 709.03. Moreover, to verify the role of these genes in the risk model, LASSO algorithm was analyzed, and the results showed that any of the five genes could not be omitted (optimal lambda value 0.0049; **Figure 4**). Based on the five genes and coefficients, we calculated each CRC patient’s risk score and divided the samples into two groups (high and low expression) according to the median risk score. After excluding patients whose survival time or status is not available (NA), only 379 CRC patients remained and K–M curves showed that the differences between the high- and low-risk groups were significant (P = 0.002; **Figure 5A**). The survival time, survival status, and gene expression of five genes in every CRC patient were vividly shown in **Figures 5E, F**.

**Table 2:** The results of univariate Cox analysis in CRC.

Gene	coefficients	HR
**ENO3**	0.317857	1.37418
**P4HA1**	0.027138	1.02751
**GPC1**	0.039966	1.040775
**SPAG4**	0.070012	1.072521
**STC2**	0.044451	1.045454

HR, hazard ratio.

### Risk Score Model

Five genes were identified and subsequently used to construct a prognostic gene signature. The risk score = 0.317856 × ENO3 + 0.027138 × P4HA1 + 0.039965 × GPC1 + 0.070012 × SPAG4 + 0.044451 × STC2, while ROC and Kaplan–Meier curves were used to assess the prognostic values of the risk scores (**Figures 5C**). The area under the ROC curves (AUCs) of the risk model were as follows: 1-year AUC: 0.606, 3-year AUC: 0.64, 5-year AUC: 0.766. Time-dependent ROC analysis showed that the risk model exhibits strong predictive ability, especially forecasting 5-year and longer OS (**Figure 5B**).

**Figure 6:**
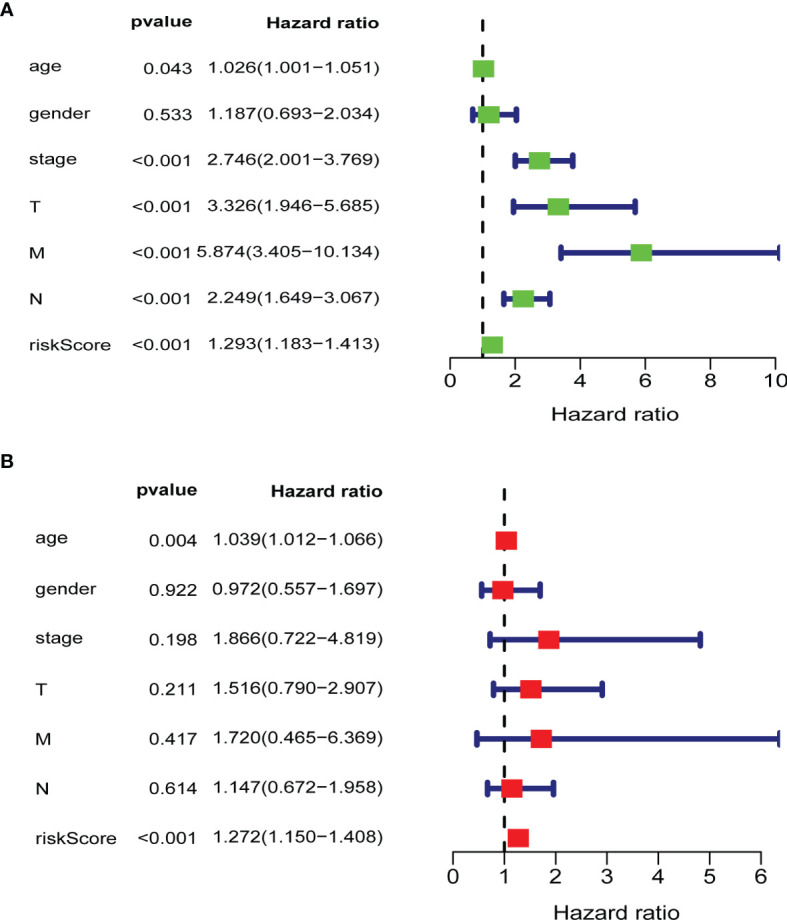
Validation of clinical independence of risk score model. **(A)** Univariable analysis for each clinical feature (age, gender, TNM stage) and risk score model. **(B)** Multivariable analysis for risk score model and clinical characteristics (age, gender, TNM stage). The green and red boxes represent the hazard ratio, and the blue bars mean 95%CI. CI, confidence interval; T, T stage; N, N stage; M, M stage; riskScore, risk score model.

To verify whether the gene signature could be an independent prognostic indicator, univariable and multivariable Cox analyses were performed. The results of the univariate analysis demonstrated that the risk score, AJCC stage, M stage, N stage, T stage, and age were significantly correlated with OS (P < 0.05; **Figure 6A**). Likewise, the results of the multivariate Cox regression analysis documented that the risk score and age were still significantly associated with the OS (P < 0.05; **Figure 6B**). Our results indicated that the glycolysis-related risk score model could be a clinically independent prognostic factor for CRC patients.

**Figure 7:**
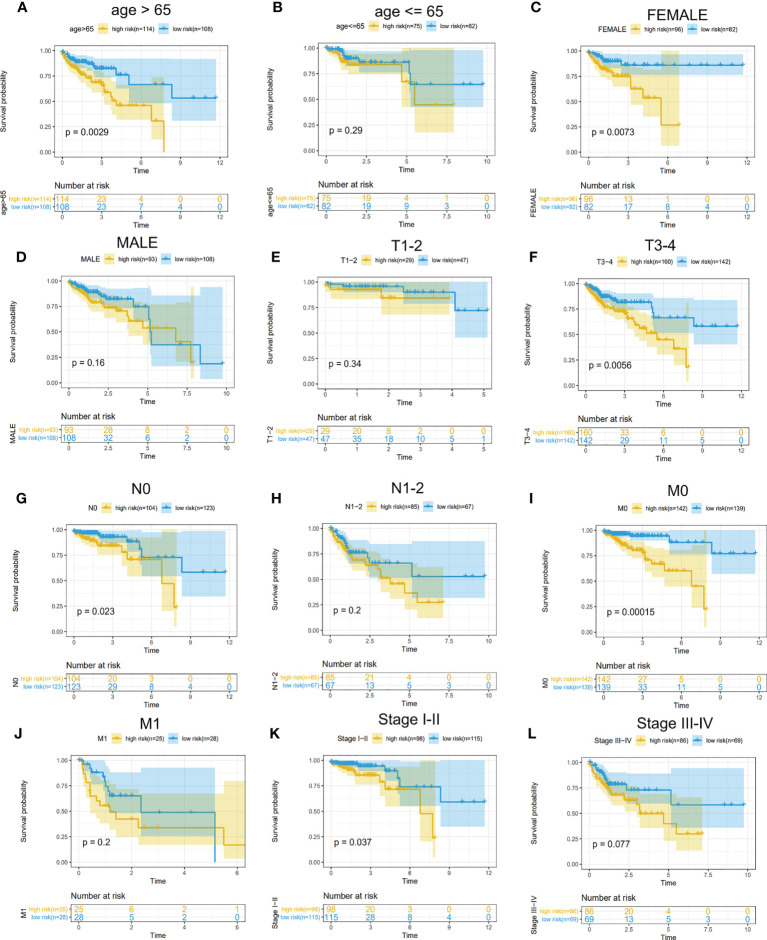
Determination of CRC patients suitable for the model. **(A–L)** Survival analysis for high- and low-risk groups in different patients. **(A, B)** Subgroups divided by age. **(C, D)** Subgroups divided by gender. **(E, F)** T1–T2 patients were divided into a common group and T3–T4 as another. **(G, H)** no lymph node metastasis (N0) as a group and lymph node-positive(N1–2) as another. **(I, J)** subgroup divided by the status of distant metastasis. **(K, L)** Stages I–II as a group and III–IV as another.

To find which groups of patients could benefit more from the risk model, we divided CRC patients into these parts: female and male, age ≤65 and age >65, T1–T2 and T3–T4 N0 and N1–2, and M0 and M1. We found that this model is more suitable for the following patients through K–M curves and log-rank test: female patients, age >65, T3–T4, N0, and M0 (**Figure 7**). We explored the different expression of these five genes in the various stages (TNM stage) and found that GPC1 and STC2 had a significant increasing trend with the advanced stage and N stage (**Figure S1**). The results suggested that GPC1 and STC2 had a close relationship with clinical metastasis in CRC patients.

### Validation of Risk Score Model in GEO Data Set

**Figure 8:**
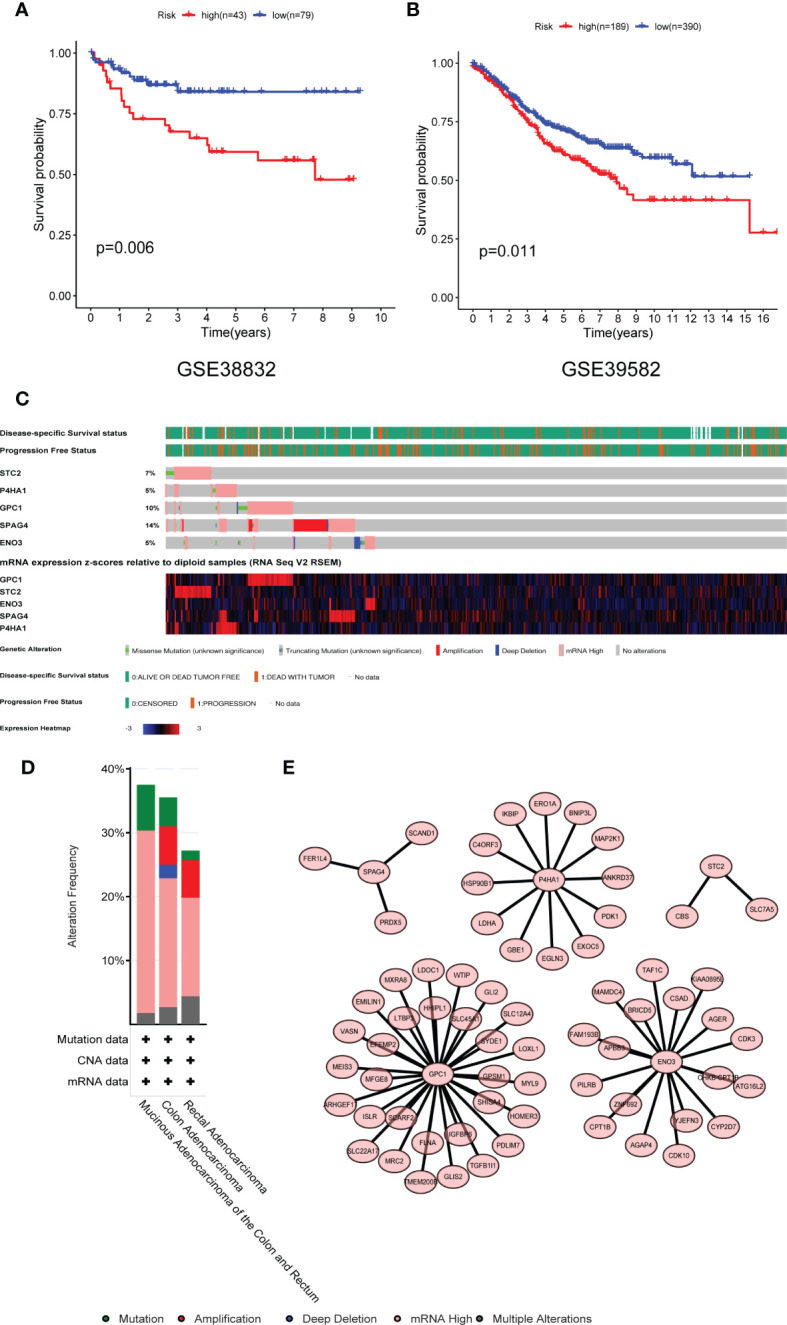
Validation of the risk model in the GEO dataset and mutational profiling of five genes. **(A)** GSE38832 and **(B)** GSE39582 dataset. **(C)** A visual summary of gene alternation from CRC. **(D)** The total alternation of five key genes. **(E)** The network of five glycolytic genes and high related expressed genes. GEO, Gene Expression Omnibus database.

In an external experimental group (GSE38832), there are 122 CRC patients, and the follow-up data was disease-free survival (DFS). Based on TCGA multivariable Cox model coefficients, every patient’s score was calculated, and the cut-off value of the risk score is 5.69 determined by Survminer R package. Hence, two subgroups (high- and low-risk groups) were separated, and the K–M curves confirmed that patients with low-risk scores had a more favorable DFS than those with high scores (*P* = 0.006; **Figure 8A**). Similarly, another external group (GSE39582) also comes from GEO with 579 CRC patients. The results also demonstrated that there were significant differences in OS between the two groups (*P* = 0.011, **Figure 8B**).

### Genetically Alternation of Five Glycolytic Genes

The mutation status of five genes was analyzed by the cBioPortal database. The five genes altered in 114 (35.54%) of 332 colon cancer patients and 37 (27.21%) of 136 rectal cancer patients (**Figure 8C**). The alternation of each gene was shown in **Figure 8D**. SPAG4 and GPC1 altered at 14 and 10%, respectively. Amplification and mRNA high were the primary mutated type. The co-expression networks of these five genes were shown in **Figure 8E**. ENO3, GPC1, and P4HA1 have more robust co-expression networks than SPAG4 and STC2.

### Build a Nomogram Based on the Risk Score Models

**Figure 9:**
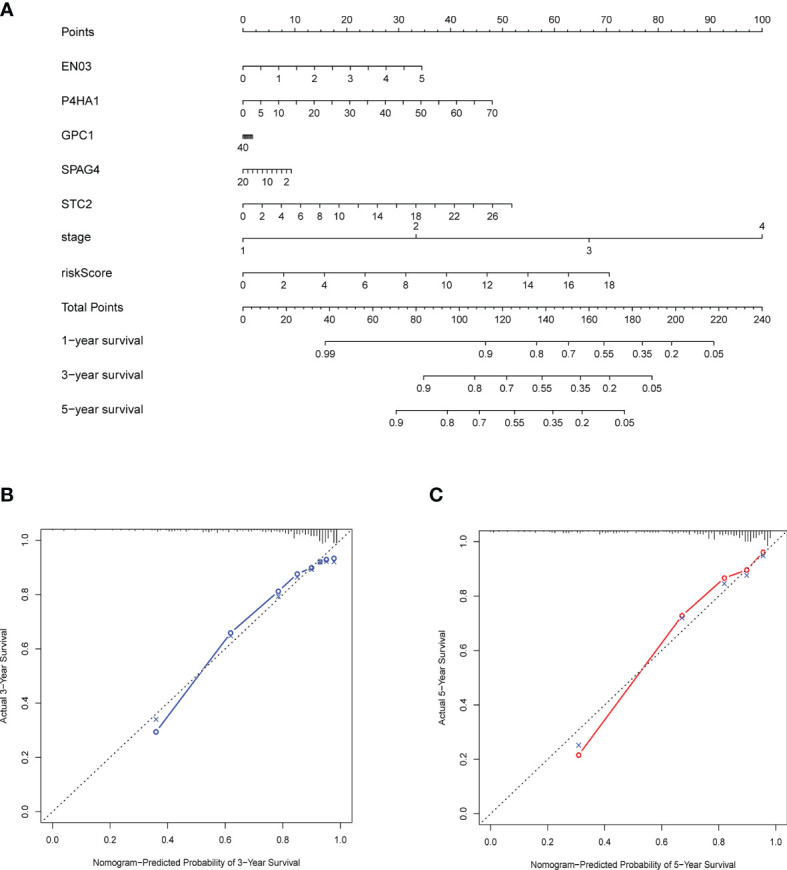
Construction and validation of a nomogram. **(A)** Nomogram with gene expression and risk model for predicting 1-, 3-, 5-year death risk. **(B, C)** Calibration curves of the nomogram to verify the agreement of predicted and actual 3-, 5-year outcomes.

For predicting 3- and 5-year OS, a nomogram was built (**Figure 9A**), and calibration and C-index were used to evaluate the discrimination and accuracy of the nomogram. In the TCGA dataset, the C-index was 0.7905. The 3- and 5-year survival probability calibration curves for the TCGA datasets demonstrated fair agreements between prediction and observation (**Figures 9B, C****)**.

### Validation of Different Expression in Protein and mRNA Levels

**Figure 10:**
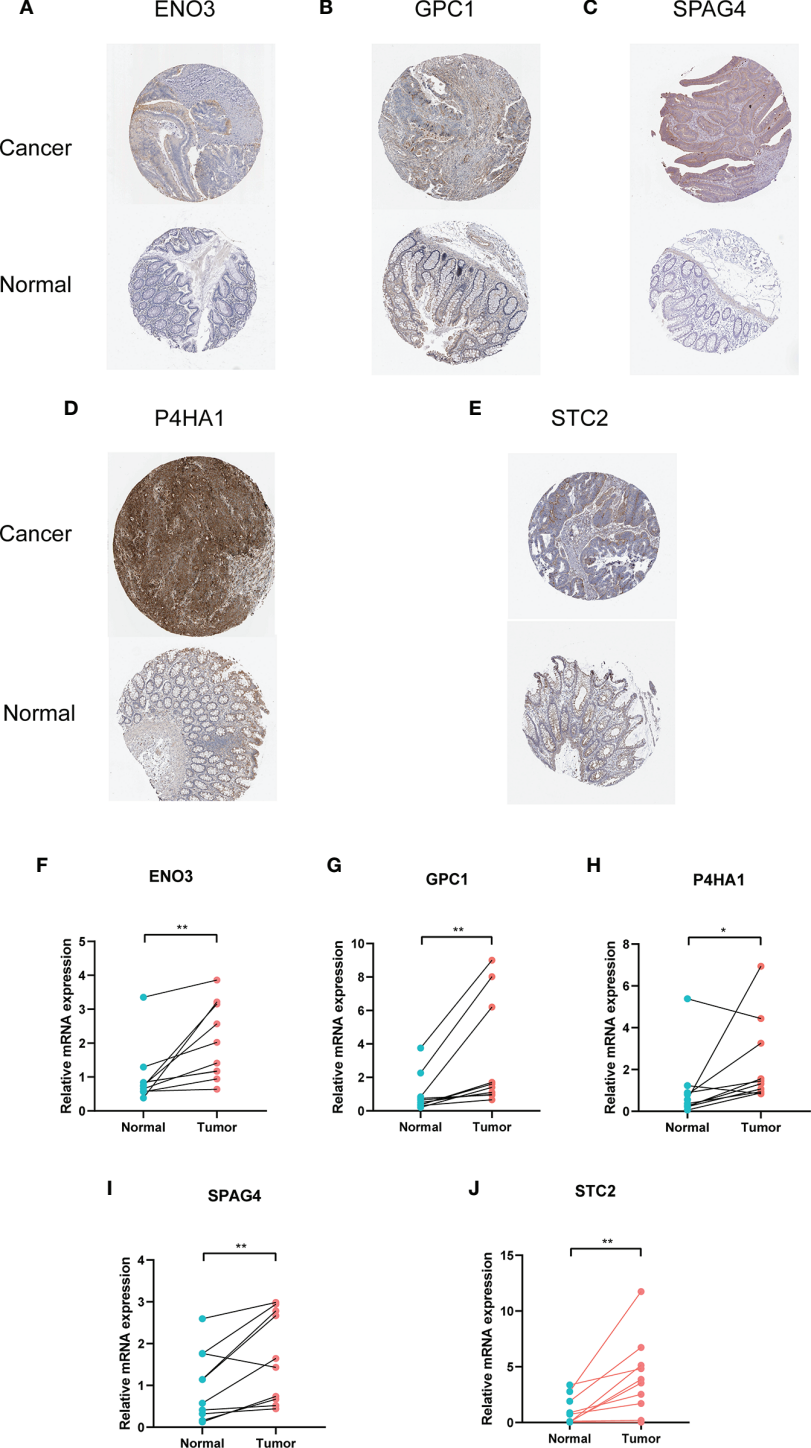
Immunohistochemistry and mRNA of the five genes by HPA database and RT-PCR. **(A)** ENO3, **(B)** GPC1, **(C)** SPAG4, **(D)** P4HA1, **(E)** STC2. **(F–J)** Relative mRNA expression of five genes in cancerous and paracancerous tissue. * means P < 0.05 and ** represents P < 0.01.

HPA database is the famous database to detect protein expression in various cancers (26). We downloaded the pictures of IHC, and the results showed the protein expression of five genes was highly upregulated in cancer tissues than in normal intestinal tissue (**Figures 10A–E**). Moreover, mRNA expression from my collected 10 CRC patients revealed the same results as the HPA database (**Figures 10 F–J**). In the *in vitro* experiments, we used a normal colorectal cell (NCM460) and five CRC cells (CACO2, HCT116, SW620, SW480 and HT29). The results of RT-PCR indicated that GPC1, STC2, P4HA4, and ENO3 were upregulated in cancer cells while SPAG4 was downregulated in the five cancer cells (**Figure S2**).

## Discussion

Recent developments in energy metabolism have led to a renewed interest in a deeper understanding of malignant tumors. Moreover, the interpretation of cancer as a genetic disease has gradually been displaced by a metabolic disease (27). The unique metabolic phenotype of cancer cells was the “Warburg effect” which revealed that even in the presence of oxygen, cancer cells preferentially shift their metabolism toward glycolysis followed by lactic acid fermentation rather than oxidative phosphorylation (OXPHOS). Accumulating evidence suggests that most CRC cells demonstrate the “Warburg” metabolic phenotype, which produces ATP more rapidly than OXPHOS. Besides, numerous glycolysis-related genes and proteins have been demonstrated to be upregulated in CRC (11–18) and correlated with tumor aggressiveness and poor prognosis (21, 28). Here, we analyzed glycolysis-related genes using a series of bioinformatics analysis and constructed a risk model based on glycolysis by multivariate and LASSO Cox regression to identify a novel biomarker for CRC patients. The model could well predict CRC patients’ prognosis and be of vital importance for the diagnosis and treatment of patients clinically.

GESA indicated that four gene clusters were enriched in cancer tissue compared with adjacent normal samples. Then, DEGs between cancer and normal samples were conducted using LIMMA method, and 236 genes were verified. The risk model by multivariate and LASSO analyses contains five genes (ENO3, GPC1, P4HA1, SPAG4, and STC2). K–M and ROC curves showed that survival time in the high-risk group is significantly shorter than in the low-risk group, and the 5-year OS predicted by this model is more reliable than 1- or 3-year OS. Enolase (ENO), also known as phosphohydrolase dehydratase, is a metalloenzyme that catalyzes the transformation of 2-phosphoglycerate to phosphoenolpyruvate during glycolysis (29). Recent researchers found the knockdown of ENO3 exhibits anticancer effect in STK11 mutant cells and suggest that ENO3-based targeted therapy might be promising for lung cancer patients harboring STK11 mutations (30). GPC1 is a membrane-anchored protein overexpressed in multiple tumors and involved in the tumorigenesis of certain cancers, including breast cancer and pancreas cancer (31, 32). With the development of exosomes in caner study, GPC1 has been a resurgence of interest because it is a cancer exosomes specific protein (33). GPC1+ circulating exosomes were regarded as a therapeutic target (34) and could facilitate the early detection of cancer (33). Another study demonstrated that combined detection of exosomal GPC1, exosomal CD82, and serum CA19-9 shows excellent promise as a standard method for pancreatic cancer detection (35). Likewise, P4HA1 is the active catalytic component of prolyl 4-hydroxylase and has been reported to promote tumor progression and metastasis in several cancers (36–38). Gaofeng Xiong et al. suggested that P4HA1 promotes chemoresistance by modulating HIF-1-dependent cancer cell stemness, and targeting collagen P4H is a promising strategy to inhibit tumor progression and sensitize TNBC to chemotherapeutic agents (39). STC2 is a glycoprotein hormone and regulates malignant tumor progression (40–42), which could be a useful biomarker for survival prediction (43). The last validated gene (SPAG4) plays a vital role in spermatogenesis and sperm motility and is a mediating protein between the nucleoskeleton and cytoskeleton (44). SPAG4 promotes survival of cancer cells under hypoxic conditions and leads to poor prognosis of several cancers, including renal cell carcinoma (45), glioblastoma (46), Pancreatic Ductal Adenocarcinoma (47). The above literature fully demonstrates that all of the selected genes are upregulated in cancer tissues. We used a statistical algorithm to construct a gene model that comprehensively integrated each gene’s prediction effect to enhance prediction efficiency.

Considering that the mRNA level of five genes was upregulated in cancerous tissues, we adopted immunohistochemical images of CRC tissues in the HPA database. The results were consistent with the variability of mRNA, which confirmed that the expression level of these proteins in cancer tissues was significantly higher than that in adjacent tissues. Moreover, based on basic clinical features, different subgroups were divided to verify the risk model’s prediction. Old female patients with early TNM stage could be predicted more precisely than young male patients with advanced stage. Another research showed that CRC did not have a strong relationship with glycolysis (48), which might be explained by the fact that they selected only three glycolysis-related gene sets and had no results of multivariate Cox regression.

Nevertheless, the present study has some limitations. Firstly, these five genes are still not reported to be key genes in the glycolysis pathway, and the underlying mechanism needs to be explored. Second, many patients had zero survival days and were regarded as one day survival in our study, which could have biased the results. In the future, we need large cohorts and basic experiments for further exploring the potential mechanism of these glycolysis-related genes.

In the current study, we provide novel insights into the relationship between glycolysis and CRC and established a glycolysis-related gene signature that could be applied to analyze patients’ prognosis with CRC. Furthermore, the nomogram models provided an insightful and applicative tool to evaluate CRC prognosis. This signature could be a promising therapeutic target in CRC patients with poor prognoses.

## Data Availability Statement

The original contributions presented in the study are included in the article/supplementary material. Further inquiries can be directed to the corresponding authors.

## Ethics Statement

The studies involving human participants were reviewed and approved by the Medical Ethics Committee of the First Affiliated Hospital of the Air Force Medical University. The patients/participants provided their written informed consent to participate in this study.

## Author Contributions

JL designed the study. JuZ and SW contributed to the conception of the study. HB, KW, and JH contributed significantly to analysis and manuscript preparation. JuZ and SW performed the data analyses and wrote the manuscript. JL and JiZ helped perform the analysis with constructive discussions. All authors contributed to the article and approved the submitted version.

## Funding

This work was supported in part by grants from the National Natural Science Foundation of China (81672751) and the Key Research and Development Program of Shaanxi (2019SF-010).

## Conflict of Interest

The authors declare that the research was conducted in the absence of any commercial or financial relationships that could be construed as a potential conflict of interest.

## Supplementary Material

Supplementary Figure 1 The relationship between five genes and clinical stage. **(A)** Expression of five glycolytic genes in the stage. **(B)** Expression of five glycolytic genes in the N stage.

Supplementary Figure 2 The differentially expressed of five genes in cell lines by RT-PCR. **(A)** GPC1; **(B)** STC2; **(C)** P4HA4; **(D)** SPAG4; **(E)** ENO3. NCM460: a kind of normal colorectal cells. CACO2, HCT116, SW620, SW480, HT29: five common colorectal cancer cells.
