# Supplementary figures and images for: Identification of Five Glycolysis-Related Gene Signature and Risk Score Model for Colorectal Cancer
Source: Front Oncol. 2021 Mar 4;11:588811. doi: 10.3389/fonc.2021.588811 (PMC7969881; doi:10.3389/fonc.2021.588811)

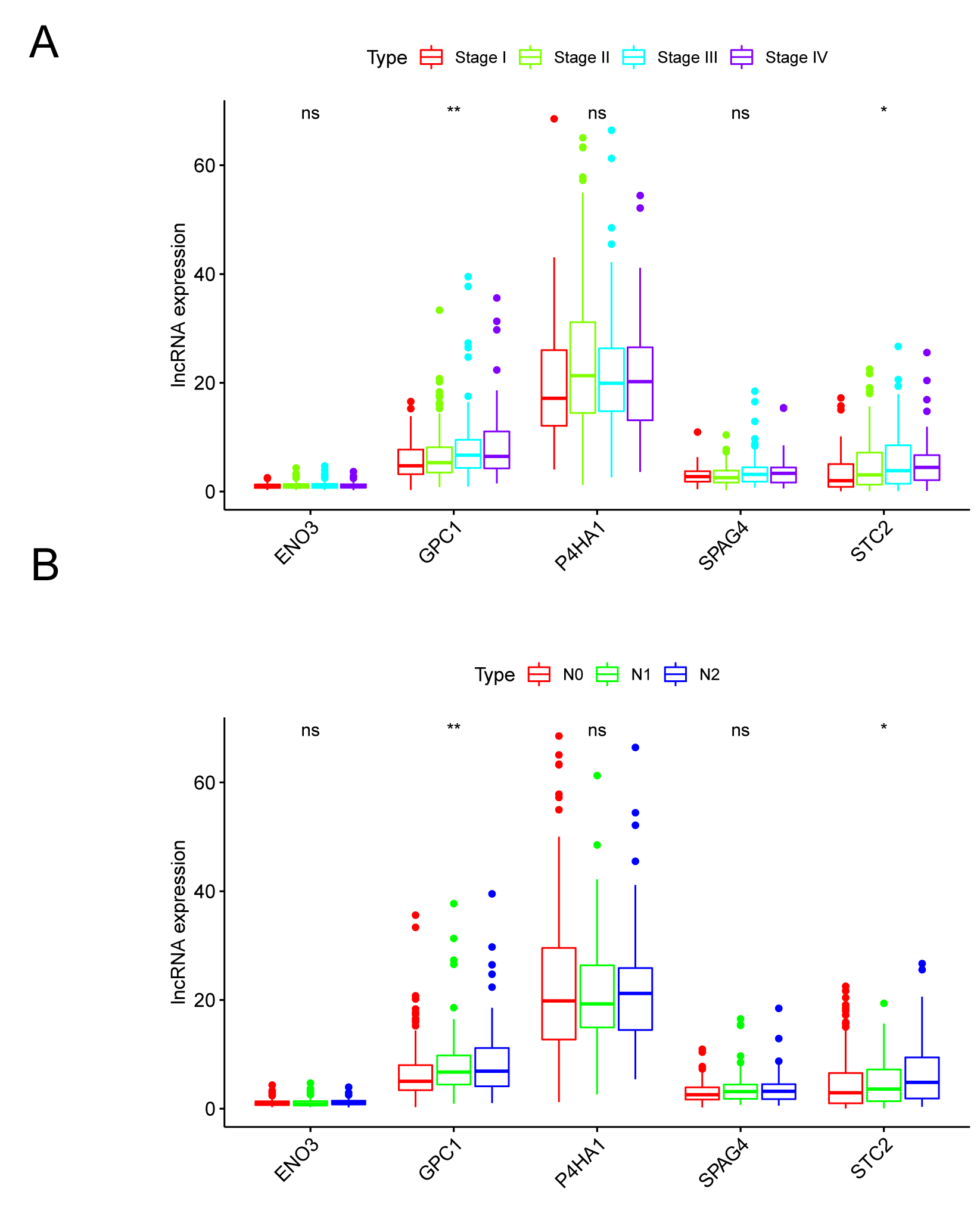

Supplement: Supplementary Figure 1 — The relationship between five genes and clinical stage. (A) Expression of five glycolytic genes in the stage. (B) Expression of five glycolytic genes in the N stage. [file Image_1.tif]

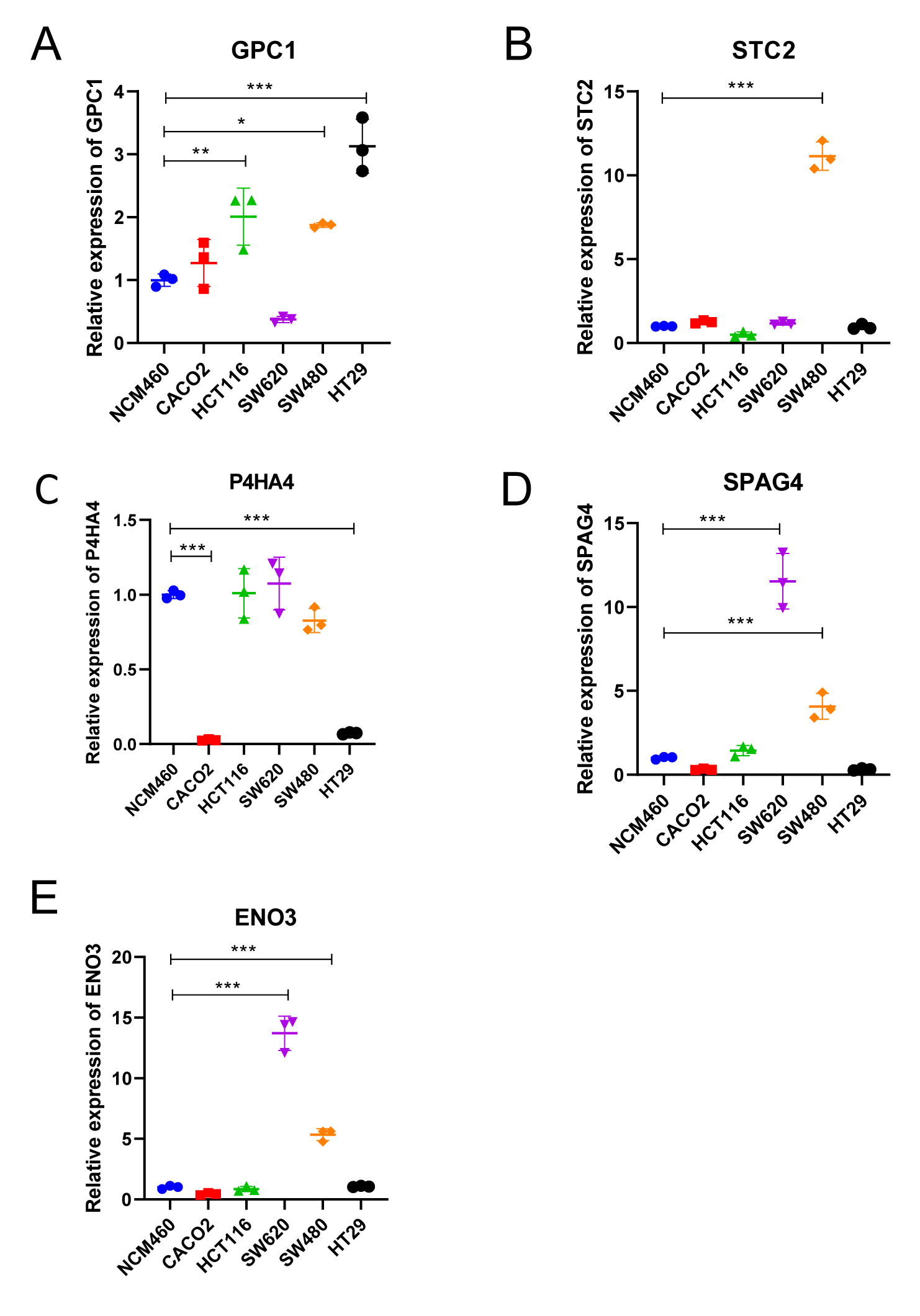

Supplement: Supplementary Figure 2 — The differentially expressed of five genes in cell lines by RT-PCR. (A) GPC1; (B) STC2; (C) P4HA4; (D) SPAG4; (E) ENO3. NCM460: a kind of normal colorectal cells. CACO2, HCT116, SW620, SW480, HT29: five common colorectal cancer cells. [file Image_2.tif]
