# Supplementary material for: Identification of Five Glycolysis-Related Gene Signature and Risk Score Model for Colorectal Cancer
Source: Front Oncol. 2021 Mar 4;11:588811. doi: 10.3389/fonc.2021.588811 (PMC7969881; doi:10.3389/fonc.2021.588811)
Supplement: Supplementary file 3 [file Table_1.docx]

**TableS1: The detailed information of the expression profile datasets**

| **Dataset** | **Array types** | **Experiment type** |
| --- | --- | --- |
| **GSE38832** | [HG-U133_Plus_2] Affymetrix Human Genome U133 Plus 2.0 Array | Expression profiling by array |
| **GSE39582** | [HG-U133_Plus_2] Affymetrix Human Genome U133 Plus 2.0 Array | Expression profiling by array |
| **The Cancer Genome Atlas (TCGA)** | Illumina HiSeq platform | RNA-sequencing profiling |
